# Supplementary material for: A Likelihood Approach for Real-Time Calibration of Stochastic Compartmental Epidemic Models
Source: PLoS Comput Biol. 2017 Jan 17;13(1):e1005257. doi: 10.1371/journal.pcbi.1005257 (PMC5240920; doi:10.1371/journal.pcbi.1005257)
Supplement: S1 File — (TAR.GZ) [file pcbi.1005257.s014.tar.gz › HSPH_Online-SI-Revision/Additions/EnKF08/n100-extreme-SEITR_noisy/n100-extreme-SEITR_noisy_EnKF2_table0.pdf]

1	newly 3 weeks spec	6225.94	2566.61	{41., 8463.}	76.8027	1.00538
2	newly 3 weeks spec	7321.	3342.44	{543., 3989.}	86.468	0.732021
3	newly 3 weeks spec	8747.	622.75	{31., 679.}	97.4566	1.78747
4	newly 3 weeks spec	8496.	2413.07	{389., 2271.}	91.3612	1.02416
5	newly 3 weeks spec	5120.	581.59	{42., 534.}	96.601	1.56305
6	newly 3 weeks spec	4778.	1185.63	{5., 361.}	106.626	1.87386
7	newly 3 weeks spec	7357.	2013.52	{72., 1953.}	100.027	1.37198
8	newly 3 weeks spec	7216.	3143.98	{368., 8478.}	82.8057	0.766312
9	newly 3 weeks spec	5164.	1659.46	{47., 1391.}	106.762	1.47722
10	newly 3 weeks spec	8694.	2559.8	{194., 10648.}	89.1898	1.12436
11	newly 3 weeks spec	8565.	425.05	{13., 1040.}	95.0374	1.85596
12	newly 3 weeks spec	7136.	2668.79	{346., 4512.}	76.6459	0.743643
13	newly 3 weeks spec	7637.	4121.68	{1087., 12549.}	74.8676	0.557024
14	newly 3 weeks spec	8364.	2094.22	{117., 6699.}	79.3556	1.07798
15	newly 3 weeks spec	7352.	1500.83	{173., 1997.}	88.0004	1.06142
16	newly 3 weeks spec	8547.	1874.8	{0., 4808.}	87.4061	1.69329
17	newly 3 weeks spec	6343.	701.93	{42., 746.}	94.1729	1.6216
18	newly 3 weeks spec	6778.	453.04	{10., 433.}	95.3202	1.78703
19	newly 3 weeks spec	8875.	1123.32	{0., 1775.}	93.3843	2.27172
20	newly 3 weeks spec	6233.	778.33	{4., 463.}	96.4917	1.96286
21	newly 3 weeks spec	6457.	837.5	{25., 504.}	99.2281	1.76691
22	newly 3 weeks spec	7395.	3771.1	{159., 14785.}	86.2529	0.875244
23	newly 3 weeks spec	4881.	4002.54	{694., 13443.}	91.3165	0.547567
24	newly 3 weeks spec	7357.	957.72	{16., 607.}	96.6301	1.84651
25	newly 3 weeks spec	9005.	511.48	{0., 1129.}	96.4433	2.47332
26	newly 3 weeks spec	6466.	1094.24	{61., 1341.}	99.2982	1.46618
27	newly 3 weeks spec	7481.	1250.18	{44., 2291.}	93.85	1.452
28	newly 3 weeks spec	5303.	990.15	{19., 397.}	102.292	1.67793
29	newly 3 weeks spec	9186.	3483.4	{668., 11590.}	80.0904	0.749808
30	newly 3 weeks spec	9408.	981.95	{0., 2601.}	90.6836	2.24303
31	newly 3 weeks spec	6334.	3506.96	{96., 10587.}	85.2469	0.957321
32	newly 3 weeks spec	8242.	3839.35	{262., 15007.}	88.0926	0.936258
33	newly 3 weeks spec	5633.	1723.36	{36., 1601.}	93.793	1.13903
34	newly 3 weeks spec	6383.	3539.11	{163., 13529.}	88.1571	0.803711
35	newly 3 weeks spec	5611.	1022.41	{23., 1248.}	98.3506	1.67793
36	newly 3 weeks spec	9648.	2708.99	{318., 7679.}	84.0316	0.90724
37	newly 3 weeks spec	8031.	2451.26	{721., 5855.}	75.7467	0.813131
38	newly 3 weeks spec	6443.	1181.57	{0., 1651.}	90.5431	1.58132
39	newly 3 weeks spec	8056.	2655.35	{161., 10312.}	84.089	0.991358
40	newly 3 weeks spec	7422.	2813.19	{393., 12104.}	85.4892	0.82506
41	newly 3 weeks spec	7711.	352.25	{8., 323.}	95.4319	1.97416
42	newly 3 weeks spec	7808.	780.69	{109., 643.}	94.8006	1.49579
43	newly 3 weeks spec	8961.	1965.1	{49., 2444.}	88.0808	1.22505
44	newly 3 weeks spec	7188.	2550.63	{269., 8372.}	83.0602	0.861719
45	newly 3 weeks spec	7528.	2563.72	{377., 2432.}	85.4134	0.802589
46	newly 3 weeks spec	8312.	1140.47	{54., 479.}	98.1017	1.77909
47	newly 3 weeks spec	8223.	369.95	{7., 1043.}	95.501	1.97828
48	newly 3 weeks spec	8405.	3653.64	{350., 18578.}	89.1695	0.814051
49	newly 3 weeks spec	6054.	3909.45	{140., 14720.}	92.8432	0.832421
50	newly 3 weeks spec	8264.	3076.98	{643., 11985.}	82.8744	0.772674

1	newly 3 weeks cumul	27440.5	8704.26	{699., 22735.}	76.5123	0.905528
2	newly 3 weeks cumul	36564.	22054.	{7734., 58011.}	73.9847	0.462616
3	newly 3 weeks cumul	40258.	3769.49	{404., 8911.}	92.6058	1.35046
4	newly 3 weeks cumul	36998.	13892.2	{3996., 21174.}	82.3102	0.69337
5	newly 3 weeks cumul	31803.	5132.25	{442., 8601.}	92.1026	1.23542
6	newly 3 weeks cumul	31868.	4658.06	{224., 6198.}	93.3382	1.49296
7	newly 3 weeks cumul	28195.	8946.82	{817., 11736.}	98.3846	1.13261
8	newly 3 weeks cumul	32722.	16208.1	{4738., 23750.}	80.1842	0.575685
9	newly 3 weeks cumul	32212.	7541.78	{610., 10641.}	93.6413	1.22147
10	newly 3 weeks cumul	33638.	18704.6	{2540., 82048.}	93.9152	0.766574
11	newly 3 weeks cumul	34772.	8941.88	{1282., 14500.}	85.7566	0.940382
12	newly 3 weeks cumul	31568.	14472.2	{2388., 19198.}	86.5815	0.710805
13	newly 3 weeks cumul	33066.	18122.8	{4724., 47474.}	79.7871	0.572669
14	newly 3 weeks cumul	30226.	5589.88	{659., 14881.}	81.5074	1.03139
15	newly 3 weeks cumul	31019.	7615.11	{1618., 27319.}	82.1189	0.89997
16	newly 3 weeks cumul	32656.	14218.	{3287., 48279.}	76.6137	0.695011
17	newly 3 weeks cumul	39322.	4721.61	{653., 4156.}	90.9158	1.33313
18	newly 3 weeks cumul	35236.	2926.82	{513., 2579.}	93.259	1.39896
19	newly 3 weeks cumul	35227.	13490.7	{3301., 47466.}	80.7001	0.772617
20	newly 3 weeks cumul	33857.	4181.13	{85., 2408.}	94.5112	1.60687
21	newly 3 weeks cumul	36224.	4134.63	{319., 7525.}	93.1352	1.3884
22	newly 3 weeks cumul	32127.	17071.9	{2229., 68439.}	85.7734	0.738632
23	newly 3 weeks cumul	26601.	19123.2	{3134., 69004.}	92.4264	0.608134
24	newly 3 weeks cumul	40276.	4857.29	{339., 2901.}	94.7981	1.49446
25	newly 3 weeks cumul	36976.	8075.8	{1281., 11074.}	89.3899	1.0532
26	newly 3 weeks cumul	27532.	5505.38	{882., 6559.}	89.2079	1.08196
27	newly 3 weeks cumul	29075.	12499.5	{859., 44005.}	93.4451	0.967368
28	newly 3 weeks cumul	34205.	5319.12	{244., 2784.}	96.2516	1.44192
29	newly 3 weeks cumul	36850.	14655.4	{3205., 35309.}	82.3107	0.737071
30	newly 3 weeks cumul	43928.	8268.99	{327., 30730.}	87.0343	1.32238
31	newly 3 weeks cumul	24601.	10672.3	{694., 44446.}	82.406	0.848321
32	newly 3 weeks cumul	32101.	19893.1	{1615., 82180.}	93.1463	0.776817
33	newly 3 weeks cumul	30340.	16003.	{2109., 42540.}	87.0055	0.741665
34	newly 3 weeks cumul	29792.	23349.9	{1819., 84017.}	96.3929	0.657468
35	newly 3 weeks cumul	36155.	6285.29	{248., 8481.}	92.1569	1.38989
36	newly 3 weeks cumul	37219.	19349.9	{2696., 62339.}	84.3718	0.669479
37	newly 3 weeks cumul	39903.	15893.1	{3552., 17552.}	88.943	0.824686
38	newly 3 weeks cumul	29746.	8946.75	{1275., 17564.}	81.583	0.82354
39	newly 3 weeks cumul	34505.	19559.9	{1562., 73837.}	87.128	0.71031
40	newly 3 weeks cumul	30770.	12849.4	{2030., 38172.}	90.3386	0.816362
41	newly 3 weeks cumul	45986.	4371.5	{534., 3611.}	94.2641	1.40698
42	newly 3 weeks cumul	27957.	3469.98	{942., 3283.}	90.3662	1.16061
43	newly 3 weeks cumul	34781.	16266.9	{737., 74897.}	95.8758	0.959521
44	newly 3 weeks cumul	31836.	12749.	{2091., 22925.}	89.7223	0.798991
45	newly 3 weeks cumul	33385.	15256.5	{4994., 21482.}	82.8521	0.61679
46	newly 3 weeks cumul	39284.	5288.6	{530., 4024.}	92.1183	1.37051
47	newly 3 weeks cumul	34349.	10546.7	{957., 16475.}	91.7169	1.01404
48	newly 3 weeks cumul	39417.	19907.8	{5004., 62689.}	85.9258	0.656303
49	newly 3 weeks cumul	27886.	19878.	{2024., 79810.}	99.906	0.742218
50	newly 3 weeks cumul	33965.	15083.6	{3083., 41941.}	88.4026	0.746127

1	newly 5 weeks spec	2726.36	2033.17	{5., 8932.}	105.018	1.20483
2	newly 5 weeks spec	2664.	1151.53	{14., 2034.}	85.0206	1.10577
3	newly 5 weeks spec	3413.	181.61	{2., 74.}	97.5678	2.34985
4	newly 5 weeks spec	3304.	690.43	{23., 1256.}	88.9676	1.33588
5	newly 5 weeks spec	1318.	151.58	{1., 117.}	98.6115	1.99576
6	newly 5 weeks spec	1057.	466.92	{0., 130.}	124.227	2.24812
7	newly 5 weeks spec	3977.	907.9	{7., 419.}	102.353	1.68983
8	newly 5 weeks spec	3068.	1246.32	{20., 2716.}	83.2471	1.0669
9	newly 5 weeks spec	1330.	430.09	{2., 262.}	109.446	1.78496
10	newly 5 weeks spec	4707.	743.48	{15., 804.}	92.1368	1.68985
11	newly 5 weeks spec	4255.	200.5	{0., 564.}	95.2879	2.8449
12	newly 5 weeks spec	3139.	1390.19	{56., 2479.}	73.9086	0.835415
13	newly 5 weeks spec	3594.	2124.8	{371., 4578.}	84.7958	0.597375
14	newly 5 weeks spec	4850.	1989.29	{31., 5761.}	71.5924	1.10889
15	newly 5 weeks spec	3380.	842.25	{21., 1283.}	89.624	1.25015
16	newly 5 weeks spec	4015.	1002.58	{0., 556.}	105.848	2.00507
17	newly 5 weeks spec	1376.	240.31	{2., 259.}	102.553	1.95945
18	newly 5 weeks spec	2583.	157.54	{0., 161.}	95.451	2.37058
19	newly 5 weeks spec	4524.	356.5	{0., 668.}	97.4297	2.52283
20	newly 5 weeks spec	2013.	246.04	{0., 194.}	97.5211	2.3762
21	newly 5 weeks spec	1848.	263.22	{1., 80.}	99.0173	2.23648
22	newly 5 weeks spec	2882.	1875.93	{9., 4882.}	90.1107	0.954677
23	newly 5 weeks spec	1370.	2032.25	{176., 8370.}	128.786	0.452868
24	newly 5 weeks spec	2182.	310.8	{0., 235.}	98.6966	2.30399
25	newly 5 weeks spec	3489.	259.84	{0., 537.}	96.9281	3.4189
26	newly 5 weeks spec	2648.	451.79	{2., 248.}	102.944	1.91445
27	newly 5 weeks spec	4125.	464.63	{0., 1032.}	95.3132	2.19714
28	newly 5 weeks spec	965.	295.61	{1., 75.}	112.816	1.92258
29	newly 5 weeks spec	4278.	1994.81	{209., 2433.}	88.0236	0.76635
30	newly 5 weeks spec	3519.	348.87	{0., 385.}	95.1768	2.59107
31	newly 5 weeks spec	3288.	2279.58	{13., 8127.}	92.528	1.09961
32	newly 5 weeks spec	4308.	1509.7	{39., 4890.}	88.1295	1.20774
33	newly 5 weeks spec	2012.	489.91	{0., 624.}	93.4001	1.80308
34	newly 5 weeks spec	2446.	1219.55	{21., 2119.}	91.5098	1.02967
35	newly 5 weeks spec	1151.	279.78	{1., 350.}	102.634	1.91224
36	newly 5 weeks spec	5029.	722.05	{34., 1054.}	88.4611	1.3054
37	newly 5 weeks spec	2840.	1113.44	{132., 1940.}	76.5944	0.816629
38	newly 5 weeks spec	2643.	303.28	{0., 471.}	95.3553	1.75065
39	newly 5 weeks spec	3317.	638.37	{5., 1077.}	90.4977	1.42949
40	newly 5 weeks spec	3674.	1393.64	{148., 1324.}	89.9733	0.848073
41	newly 5 weeks spec	1635.	64.97	{1., 60.}	96.0557	2.16748
42	newly 5 weeks spec	4650.	362.06	{6., 317.}	94.6624	1.90911
43	newly 5 weeks spec	4598.	477.21	{2., 717.}	92.4461	1.68479
44	newly 5 weeks spec	3246.	1195.23	{28., 2722.}	78.6269	0.933739
45	newly 5 weeks spec	3187.	942.18	{20., 1094.}	84.9325	1.03458
46	newly 5 weeks spec	3124.	412.34	{3., 100.}	100.761	2.32523
47	newly 5 weeks spec	3657.	138.6	{0., 543.}	96.21	3.02876
48	newly 5 weeks spec	2763.	1245.	{21., 2669.}	85.4007	0.963917
49	newly 5 weeks spec	2454.	1751.93	{11., 4934.}	93.434	0.963107
50	newly 5 weeks spec	3752.	1294.47	{213., 1518.}	85.9672	0.809088

1	newly 5 weeks cumul	34535.3	13021.3	{758., 40194.}	74.771	0.894764
2	newly 5 weeks cumul	43633.	25149.	{9068., 73736.}	75.2738	0.481742
3	newly 5 weeks cumul	49075.	4290.03	{442., 9963.}	93.5384	1.41303
4	newly 5 weeks cumul	45863.	15861.9	{4399., 21832.}	83.7065	0.731991
5	newly 5 weeks cumul	35687.	5581.28	{488., 9736.}	92.1853	1.2571
6	newly 5 weeks cumul	35197.	5857.31	{244., 6213.}	94.354	1.50442
7	newly 5 weeks cumul	37670.	11282.9	{941., 14666.}	98.3243	1.18868
8	newly 5 weeks cumul	40537.	19362.	{5739., 40305.}	78.1142	0.583258
9	newly 5 weeks cumul	36196.	8839.08	{669., 11558.}	94.9675	1.24561
10	newly 5 weeks cumul	44803.	20781.5	{2640., 96681.}	91.3876	0.838359
11	newly 5 weeks cumul	45069.	9415.11	{1389., 14501.}	84.9901	0.992453
12	newly 5 weeks cumul	39318.	17686.1	{3044., 34897.}	81.4737	0.681012
13	newly 5 weeks cumul	41882.	23118.4	{6025., 75881.}	76.1135	0.553878
14	newly 5 weeks cumul	41750.	9640.08	{892., 32603.}	77.1233	1.00067
15	newly 5 weeks cumul	39368.	9576.17	{1884., 31633.}	81.6207	0.909076
16	newly 5 weeks cumul	42788.	16550.2	{3419., 50324.}	75.564	0.748382
17	newly 5 weeks cumul	43673.	5365.54	{663., 4895.}	91.3181	1.35483
18	newly 5 weeks cumul	42014.	3351.78	{514., 2989.}	93.5625	1.43829
19	newly 5 weeks cumul	46101.	14487.8	{3436., 52116.}	80.1824	0.837625
20	newly 5 weeks cumul	39540.	4855.98	{86., 2920.}	94.6916	1.63698
21	newly 5 weeks cumul	41535.	4874.78	{357., 8598.}	93.5603	1.42203
22	newly 5 weeks cumul	39687.	21562.9	{3025., 89291.}	85.3569	0.734628
23	newly 5 weeks cumul	30613.	23898.	{4102., 79413.}	91.6525	0.57142
24	newly 5 weeks cumul	46540.	5715.11	{342., 3518.}	94.536	1.51669
25	newly 5 weeks cumul	46402.	8686.46	{1336., 11093.}	89.2957	1.1125
26	newly 5 weeks cumul	34415.	6677.33	{986., 8221.}	91.8382	1.1395
27	newly 5 weeks cumul	38835.	13716.4	{973., 46790.}	90.0703	1.02874
28	newly 5 weeks cumul	37460.	6180.39	{250., 2988.}	96.9904	1.45706
29	newly 5 weeks cumul	47405.	19259.	{3923., 61776.}	81.0413	0.722538
30	newly 5 weeks cumul	53150.	9187.48	{334., 44266.}	86.0736	1.36983
31	newly 5 weeks cumul	32650.	15784.1	{758., 64006.}	77.7902	0.823733
32	newly 5 weeks cumul	42469.	23707.8	{1885., 95659.}	89.7413	0.806534
33	newly 5 weeks cumul	35798.	17327.7	{2420., 56242.}	85.0988	0.75763
34	newly 5 weeks cumul	36225.	26516.3	{2156., 93900.}	93.3521	0.667493
35	newly 5 weeks cumul	39920.	7094.16	{261., 9462.}	92.9091	1.40896
36	newly 5 weeks cumul	49093.	21416.3	{3106., 72978.}	83.0066	0.719964
37	newly 5 weeks cumul	47625.	18537.9	{4345., 28737.}	84.7986	0.7894
38	newly 5 weeks cumul	36633.	9847.27	{1275., 18025.}	83.0602	0.867394
39	newly 5 weeks cumul	43007.	21424.1	{1601., 87818.}	85.5909	0.752254
40	newly 5 weeks cumul	39721.	16167.6	{2538., 67840.}	87.6062	0.806564
41	newly 5 weeks cumul	51212.	4583.46	{566., 3649.}	94.1887	1.43137
42	newly 5 weeks cumul	38862.	4408.01	{1112., 3898.}	91.3693	1.23977
43	newly 5 weeks cumul	46123.	17654.9	{990., 86135.}	92.0973	1.00754
44	newly 5 weeks cumul	39959.	15626.6	{2715., 39602.}	86.1736	0.785046
45	newly 5 weeks cumul	41374.	17716.7	{5851., 29012.}	81.798	0.63262
46	newly 5 weeks cumul	47611.	6388.13	{598., 4907.}	93.2113	1.43051
47	newly 5 weeks cumul	43642.	10877.6	{958., 16514.}	89.7747	1.07047
48	newly 5 weeks cumul	47174.	23246.9	{5785., 83955.}	85.3266	0.660647
49	newly 5 weeks cumul	34341.	24155.5	{2113., 93304.}	96.5007	0.734241
50	newly 5 weeks cumul	43375.	18325.2	{3994., 68996.}	85.6408	0.740219

1	newly 50 weeks cumul	41541.4	23225.2	{796., 88856.}	88.3824	0.911969
2	newly 58 weeks cumul	47260.	27010.1	{11480., 78039.}	74.9788	0.481228
3	newly 60 weeks cumul	54208.	4540.62	{463., 9996.}	93.8042	1.44914
4	newly 58 weeks cumul	50745.	16794.4	{4423., 22062.}	83.6417	0.746508
5	newly 60 weeks cumul	37045.	5735.14	{508., 10229.}	92.0755	1.2649
6	newly 59 weeks cumul	35989.	6586.71	{263., 6213.}	95.819	1.50444
7	newly 51 weeks cumul	47209.	12752.5	{1036., 16294.}	96.8102	1.23159
8	newly 58 weeks cumul	46063.	22372.	{7072., 62786.}	76.6796	0.588998
9	newly 60 weeks cumul	37584.	9303.53	{673., 11789.}	95.2652	1.25408
10	newly 56 weeks cumul	56292.	21888.7	{2663., 105047.}	88.9391	0.903634
11	newly 55 weeks cumul	54734.	10104.4	{1497., 15821.}	84.3475	1.0179
12	newly 56 weeks cumul	44760.	23448.3	{4501., 70542.}	75.327	0.597004
13	newly 57 weeks cumul	49021.	29316.9	{7154., 97074.}	75.778	0.527479
14	newly 50 weeks cumul	54343.	23659.9	{923., 86540.}	76.0176	0.960257
15	newly 51 weeks cumul	45493.	12462.1	{2028., 37372.}	79.0935	0.882122
16	newly 54 weeks cumul	49793.	18047.9	{3450., 51968.}	76.5437	0.790117
17	newly 60 weeks cumul	44678.	5727.45	{663., 5297.}	91.4531	1.35726
18	newly 59 weeks cumul	45747.	3607.51	{514., 3128.}	93.5365	1.45821
19	newly 54 weeks cumul	56361.	15253.7	{3469., 52212.}	79.5916	0.887867
20	newly 59 weeks cumul	42220.	5250.12	{86., 3388.}	94.7031	1.64626
21	newly 60 weeks cumul	43675.	5318.55	{368., 8918.}	93.2791	1.43237
22	newly 56 weeks cumul	44378.	27677.1	{3279., 100743.}	87.3432	0.691636
23	newly 57 weeks cumul	31913.	29693.7	{5192., 107005.}	95.7226	0.5236
24	newly 59 weeks cumul	48960.	6114.29	{342., 3946.}	94.3682	1.51997
25	newly 55 weeks cumul	51783.	9682.16	{1347., 14035.}	88.7241	1.1174
26	newly 53 weeks cumul	38357.	7332.79	{1023., 9312.}	92.9711	1.17004
27	newly 53 weeks cumul	49190.	14509.5	{999., 47326.}	87.1674	1.08638
28	newly 60 weeks cumul	38276.	6505.92	{250., 3014.}	97.0912	1.45955
29	newly 57 weeks cumul	55913.	25606.2	{4452., 89059.}	78.7447	0.653934
30	newly 60 weeks cumul	58223.	9751.57	{334., 45367.}	85.4424	1.39439
31	newly 50 weeks cumul	39652.	27735.8	{819., 86242.}	88.7896	0.811393
32	newly 56 weeks cumul	52787.	27913.	{2008., 100854.}	86.0165	0.815956
33	newly 60 weeks cumul	38479.	18375.2	{2499., 63171.}	83.6335	0.751659
34	newly 56 weeks cumul	39694.	29473.5	{2539., 100387.}	90.7643	0.638012
35	newly 60 weeks cumul	40865.	7456.77	{261., 11045.}	93.1373	1.41136
36	newly 56 weeks cumul	60543.	22686.3	{3216., 74961.}	81.2443	0.756508
37	newly 57 weeks cumul	51821.	22650.	{4744., 79704.}	80.1841	0.728338
38	newly 54 weeks cumul	40381.	10210.5	{1399., 18068.}	83.5677	0.886548
39	newly 56 weeks cumul	48356.	22494.	{1608., 93429.}	84.4585	0.770721
40	newly 57 weeks cumul	48338.	20944.8	{2740., 84741.}	85.3754	0.777521
41	newly 60 weeks cumul	52636.	4646.41	{573., 3649.}	94.143	1.43751
42	newly 51 weeks cumul	51353.	5147.6	{1223., 4763.}	91.3742	1.31587
43	newly 56 weeks cumul	56656.	18821.5	{1026., 86142.}	88.561	1.02856
44	newly 56 weeks cumul	46072.	19826.	{3290., 90030.}	82.4589	0.72781
45	newly 58 weeks cumul	46827.	19489.2	{6232., 29096.}	80.9756	0.635853
46	newly 60 weeks cumul	52255.	6926.78	{625., 5475.}	93.4918	1.46257
47	newly 55 weeks cumul	49521.	11385.8	{958., 19076.}	88.301	1.08083
48	newly 58 weeks cumul	50734.	25366.9	{5785., 101255.}	84.223	0.643587
49	newly 56 weeks cumul	38035.	29660.2	{2119., 99791.}	95.2457	0.685896
50	newly 57 weeks cumul	50179.	21706.3	{4726., 88011.}	81.7187	0.696448
